# Post-craniotomy intracranial pressure monitoring: a novel approach combining optic nerve sheath diameter ultrasonography and cervical-cerebral arterial ultrasound

**DOI:** 10.3389/fneur.2024.1472494

**Published:** 2025-01-15

**Authors:** Zunfeng Fu, Lin Peng, Laicai Guo, Naixia Hu, Yamin Zhu, Shouxin Tang, Hongliang Lou, Jiajun Zhang, Chongqiang Wang

**Affiliations:** ^1^Department of Ultrasound, The Second Affiliated Hospital of Shandong First Medical University, Tai'an, China; ^2^Department of General Practice, The Second Affiliated Hospital of Shandong First Medical University, Tai'an, China; ^3^Department of Neuro-Intensive Care Unit, The Second Affiliated Hospital of Shandong First Medical University, Tai'an, China; ^4^Department of Ultrasound, Dongping County People's Hospital, Tai'an, China

**Keywords:** ultrasonography, optic nerve sheath diameter, intracranial pressure, traumatic brain injury, noninvasive monitoring, transcranial color doppler

## Abstract

**Objective:**

Elevated intracranial pressure (ICP), a common complication in traumatic brain injuries (TBI), can lead to optic nerve sheath diameter (ONSD) enlargement and flow spectrum changes from the internal carotid artery (ICA) to middle cerebral artery (MCA). This study will investigate the use of Cervical-Cerebral Arterial Ultrasound (CCAU) for non-invasive ICP assessment and evaluating the related indices’ clinical utility in TBI patients with decompressive craniotomy (DC).

**Methods:**

ONSD and flow spectrum changes were measured within 24 h after DC in 106 patients via ultrasonic ONSD measurement and CCAU, simultaneously. Intracranial pressures were invasively monitored, using a microsensor or ventricular catheter as the gold standard. Patients were classified into two groups, namely the normal group and the elevated group, based on distinct intracranial pressure thresholds of 15 mmHg, 20 mmHg and 22 mmHg. Subsequently, Bland Altman plot used for evaluating agreement between estimate for ICP (ICPe) and invasive ICP (ICPi). Then, the correlation between ONSD, MCAPI (pulsatility index of MCA), PI-ratio (MCAPI/ICAPI), and ICP_e_ was examined through linear regression analysis. Finally, receiver operator characteristic curves (ROC) were also analyzed for different indexes and their combinations (using logistic model).

**Results:**

Significant differences were observed between the normal and elevated ICP groups with respect to ONSD, PI-ratio, MCAPI and MCAFVd (diastolic flow velocity of MCA) (*p* < 0.05). The correlation coefficients for the relationships between ONSD, PI ratio, FVd_MCA_, and PI with ICPi were 0.62, 0.33, 0.32 and 0.21, respectively, each demonstrating statistical significance (*p* < 0.05). Analysis of the ROC curves demonstrated that the area under the curve (AUC) for predicting elevated ICPi at thresholds of 15 mmHg, 20 mmHg, and 22 mmHg via combined ultrasonographic measurements of the PI ratio and ONSD was the largest, specifically 0.74 (95% CI: 0.65–0.82), 0.77 (95% CI: 0.69–0.85), and 0.79 (95% CI: 0.70–0.86), respectively.

**Conclusion:**

Ultrasonographic measurements of ONSD, PI-ratio, MCAPI and MCAFVd demonstrate a moderate to low weak correlation with ICPi measurements. ICPe is not considered sufficiently precise for noninvasive accurate ICP assessment. The concurrent utilization of CCAU and ONSD measurements may offer superior accuracy for elevated ICP in TBI patients with DC, especially in specificity. Further research is imperative to validate these findings within a more extensive patient population.

## Introduction

The quantification and monitoring of intracranial pressure (ICP) are considered standard care for severe Traumatic Brain Injuries (TBI) patients, suspected of having intracranial hypertension (1). Within fluid-based systems, external ventricular drainage (EVD) is commonly acknowledged as the reference standard. Similarly, the accuracy of microtransducers in monitoring ICP rivals that of EVDs (2). However, these invasive ICP (ICPi) monitorings can lead to several complications, such as infection, hemorrhage, catheter obstruction and parenchymal brain injury (1, 3), and may not be readily available in all medical settings for their expensive costs and technical requirements.

For patients with TBI who have undergone decompressive craniotomy (DC), the utility of ICP monitoring is a subject of debate. Advocates for ICP monitoring assert that sustaining an adequate cerebral perfusion pressure (CPP) is crucial for TBI management, and that without it, formulating and executing CPP-directed treatment plans becomes difficult. Research indicates that a considerable number of TBI patients still face elevated ICP post-DC, which could result in a dangerously low CPP (<60 mmHg). As a result, ICP monitoring is considered indispensable for these patients, providing a vital basis for treatment decisions informed by CPP (4, 5). Conversely, some scholars oppose the routine use of ICP monitoring. They argue that skilled clinicians can approximate ICP levels by gaging the pressure at the decompression window and by using CT or MRI to identify recurrent hematomas, effusions, or changes in ventricular size and shape, thus allowing for a comprehensive assessment of ICP. Additionally, they point out that ICP monitoring can increase patient costs and the risk of complications. There is also a lack of consensus on the critical ICP thresholds following DC, and for patients with severe TBI, care focused on maintaining monitored ICP at 20 mmHg or less was not shown to be superior to care based on imaging and clinical examination (6, 7). Despite the differences in the two aforementioned viewpoints, neither side denies the significance of ICP monitoring. Instead, they emphasize the importance of correctly applying the information provided by monitoring and the importance of non-invasive ICP monitoring as an alternative (8).

In contemporary practice, non-invasive techniques such as optic nerve sheath diameter (ONSD) assessment, pupillometry, transcranial Doppler (TCD), HeadSense technology, Flash Visual Evoked Potential (FVEP), and multimodal approaches are utilized to evaluate ICP. Previous studies (9, 10) have demonstrated that pupillometry does not significantly correlate with ICP. Although HeadSense monitors and FVEP methods have shown promising correlations, there is still a need for precise data on their sensitivity and specificity (11, 12). At present, ONSD and TCD have exhibited fair to good accuracy when compared with ICP measurements (1). In our study, we have also explored the value of these two methodologies. Naturally, we have chosen the Transcranial Color Doppler (TCCD) technique over the TCD blind method, capitalizing on TCCD’s capacity to deliver more precise hemodynamic insights.

On the other hand, we have introduced the concept of Cervical-Cerebral Arterial Ultrasound (CCAU) in our study, which provides insights into the relative changes in blood flow spectrum parameters from ICA (the internal carotid artery) to MCA (the middle cerebral artery). Normally, the pulse index (PI) of the middle cerebral artery (MCAPI) is anticipated to be lower than that of the internal carotid artery (ICAPI), and the theoretical PI-ratio (MCAPI/ICAPI) is less than 1. However, in cases of elevated ICP, the PI-ratio becomes higher because the MCAPI exhibits a more pronounced increase in order to adapt to the elevation of ICP. Theoretically, the inclusion of ICA spectral information enhances specificity of detecting intracranial hypertension, making PI-ratio more specific than MCAPI alone. Therefore, this study also delves into the clinical significance of the PI-ratio ascertained by CCAU in patients with DC.

In this research, we strived to probe the correlation between the ultrasonographic parameters of ONSD and TCCD and ICPi monitoring among patients with DC. Additionally, we evaluated the feasibility and clinical applicability of ONSD, TCCD, and CCAU in detecting elevated ICP in such patients.

## Methods

### Populations

This prospective observational study was conducted at the Department of Neuro-intensive Care Unit (NICU) of the second affiliated hospital of the First Medical University of Shandong, in Taian City, Shandong Province, China. The study was carried out from May 2021 to December 2023 and received approval from the Ethics Committee of the second affiliated hospital of the First Medical University of Shandong (2021-A-037). Informed consent was obtained from the legal guardians of all participants. All research methods were performed in accordance with the relevant guidelines and regulations.

In this study, adult patients aged 18 years or older with TBI who underwent primary DC and required ICP monitoring were enrolled within 24 h post-surgery. Exclusion Criteria: Patients were excluded if they had poor TCCD window quality affecting TCCD 2D images, significant cardiac or vascular diseases like severe arrhythmias, critical valvular stenosis or moderate to severe cerebral vasospasm, or ICPi fluctuations over 10 mmHg within 24 h.

The data collected included: the initial Glasgow Coma Scale (GCS) score upon admission, patient’s age and gender, ultrasongraphic ONSD measurements and other indexes obtained from ultrasound, length of stay in NICU, and mortality rate at the time of discharge (details in Table 1).

**Table 1:** Characteristics of the patients included in our cohort.

Parameters	TBI with intracranial hypertension (*n* = 34)	TBI with no intracranial hypertension (*n* = 72)	*p* value
Demographics			
Age, years	59 [54–66]	63 [54–67]	0.302
Male, *n* (%)	24 (70.59%)	45 (62.50%)	0.417
BMI (kg/m^2^)	24.42 [22.86–26.41]	25.76 [24.13–28.16]	0.162
NICU length of stay (days)	17 [13–24]	16 [12–24]	0.231
GCS on admission	7 [5–11]	6 [4–10]	0.165
hours from ICPi to US assessment	12 [8–14]	12 [8–13]	0.862
Comorbidities			
Diabetes, *n* (%)	4 (11.76%)	15 (20.83%)	0.258
COPD/asthma, *n* (%)	6 (17.65%)	6 (8.33%)	0.160
Liver cirrhosis, *n* (%)	4 (11.76%)	2 (2.78%)	0.063
Cancer, *n* (%)	1 (2.94%)	4 (5.56%)	0.555
Within 24 h after ICPi			
Vasopressors, *n* (%)	6 (17.65%)	11 (15.28%)	0.758
GCS	6 [3–10]	6 [3–8]	0.388
Invasive and non-invasive ICP related indexes			
ICPe, mm Hg^*^	23.1 [15.9–33.4]	16.8 [8.3–24.8]	0.012
24 h averaged ICPi, mm Hg^*^	22 [21–27]	13 [11–15]	0.000
MAP, mm Hg	90 [85–95]	86 [81–90]	0.064
Mean ONSD, mm^*^	5.0 [4.3–5.3]	4.6 [4.3–4.8]	0.006
Mean ONSD(T), mm^*^	5.2 [4.5–5.3]	4.6 [4.4–4.8]	0.000
ICAPI	1.1 [1.0–1.2]	1.1 [0.90–1.3]	0.457
MCAPI_*_	1.1 [0.9–1.2]	1.0 [0.8–1.1]	0.009
PI-ratio^*^	1.05 [0.93–1.18]	0.93 [0.83–0.97]	0.001
ICAFVs, cm/s	59 [50–95]	79 [66–91]	0.230
ICAFVm, cm/s	35 [28–51]	46 [34–51]	0.158
ICAFVd, cm/s	23 [17–32]	28 [21–32]	0.247
MCAFVs, cm/s	99 [74–122]	108 [89–126]	0.631
MCAFVm, cm/s	50 [44–61]	59 [45–74]	0.172
MCAFVd, cm/s^*^	31 [24–39]	37 [29–47]	0.006
FVs-ratio	1.50 [1.27–1.80]	1.40 [1.15–1.71]	0.410
FVm-ratio	1.45 [1.22–1.85]	1.41 [1.05–1.72]	0.821
FVd-ratio	1.53 [1.26–1.79]	1.50 [1.17–1.88]	0.709
Morbidity			
NICU mortality, *n* (%)^*^	7 (20.59%)	3 (4.17%)	0.007
Reasons for DC			
Acute subdural hematoma	61 (57.55%)		
Acute Intracerebral hematoma	29 (27.36%)		
Cerebral contusion/lacer-ation	16 (15.09%)		

NICU, neuro-intensive care unit; GCS, Glasgow Coma Scale; COPD, chronic obstructive pulmonary disease; ONSD, optic nerve sheath diameter for averaged two eyes; PI, pulsatility index; ICP, intracranial pressure; ICPe, estimate for intracranial pressure; MAP, mean arterial pressure; ONSD(T), transverse diameter of optic nerve sheath; FVd, diastolic flow velocity; FVm, mean flow velocity; FVs, systolic flow velocity; ICA, internal carotid artery; MCA, middle cerebral artery; ICAFVs, systolic flow velocity of internal carotid artery; ICAFVm, mean flow velocity of internal carotid artery; ICAFVd, diastolic flow velocity of internal carotid artery; MCAFVs, systolic flow velocity of internal carotid artery; MCAFVm, mean flow velocity of internal carotid artery; MCAFVd, diastolic flow velocity of middle cerebral artery; FVs-ratio, MCAFVs/MCAFVs; FVm-ratio, MCAFVm/ICAFVm; FVd-ratio, MCAFVd/ICAFVd; DC, decompressive craniotomy.

*, *p* < 0.05 vs. TBI with no intracranial hypertension.

### Treatment methods

In patients with TBI, the surgeon determines whether to perform DC based on the mechanism of injury, clinical presentation, GCS scores, and computed tomography (CT) results. For hematomas localized to a single cerebral hemisphere, we perform unilateral DC; in cases of bilateral hematomas or frontal lobe injuries, bilateral or frontotemporal decompression is chosen. DC is also implemented for patients with diffuse brain injury. The surgical incision, as needed, typically employs a bone window of (9 × 9 cm), and the incision is extended in an arcuate manner to the bone window and the edge of the dura mater. Hematomas, necrotic brain tissue, and blood clots are excised to ensure effective hemostasis. Subsequently, instead of a watertight closure, the remaining dura mater or artificial dura substitutes are used to loosely cover the brain surface. ICP was measured using either an intraparenchymal probe (Codman & Shurtleff, MA, USA) or a ventricular catheter connected to an external pressure transducer and drainage system (Codman, Johnson & Johnson Medical Ltd., Raynham, MA). The placement of the ICP monitoring probe was determined by lesion location, being ipsilateral to a unilateral lesion and on the right side for diffuse injury.

Monitoring and management of ICP were guided by a protocol-driven approach, involving sedation, optimizing CPP, administering hyperosmolar fluids, hypothermia, in accordance with institutional guidelines.

### ONSD measurements

To reduce the impact of subjective biases that could occur when a single expert performs duplicate measurements on the same patient within a short period, we collected the relevant ONSD data by two different ultrasound experts (Y. Zhu with 8 years of experience in vascular ultrasound and S. Tang with 3 years of experience in TCCD) within 24 h after DC (The collection times were at the 6th hour and 18th hour after the operation). These experts had been trained in the “Color Doppler - Low power examination - Optic disk clarity - Safety [short examination duration] - Elevate frequency - Dual measurements” (CLOSED) protocol proposed by Aspide et al. (13). The average value of the two sets of measurements was used as the final measurement value in the study.

**Figure 1:**
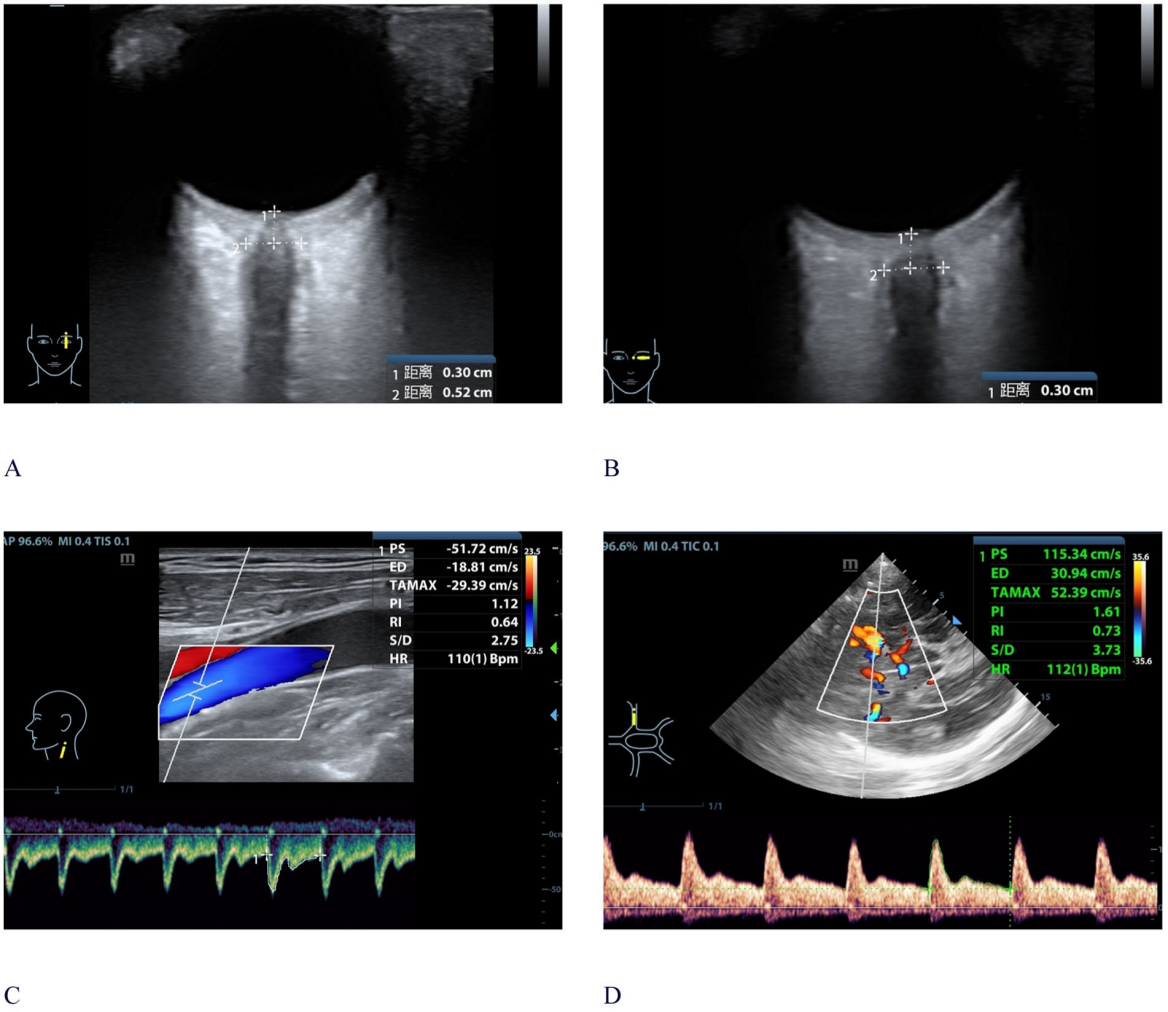
Bedside ultrasonographic measurements of CCAU and ONSD. **(A)** ONSD ultrasonography in the sagittal plane; **(B)** ONSD ultrasonography in the transverse plane; **(C)** CCAU measurement of ICA; **(D)** CCAU measurement of MCA. A 59-year-old male patient with a 24 h averaged ICPi of 23.6 mmHg, monitored following DC. CCAU and ONSD measurement were conducted within 24 h after ICPi. The ONSD value was 5.2 mm, with ICAPI: 1.12 and MCAPI: 1.61. The PI-ratio was greater than 1. CCAU, Cervical-Cerebral Arterial Ultrasound; ONSD, optic nerve sheath diameter; DC, decompressive craniotomy; ICPi_,_ invasive intracranial pressure; PI, pulsatility index; MCAPI_,_ pulsatility index of middle cerebral artery; ICAPI_,_ pulsatility index of internal carotid artery; PI-ratio, MCAPI/ICAPI; ICA, internal carotid artery; MCA, middle cerebral artery.

During the ONSD measurement procedure, the Mindray M9 ultrasound system (Shenzhen, Guangzhou, China) equipped with an L12 - 4 s (4–12 MHz) probe was employed. In accordance with safety guidelines, the output of the ultrasonic equipment was calibrated based on the “as low as reasonably achievable” (ALARA) principle (13). According to established protocols, ONSD measurements were recorded at a depth of 3 mm posterior to the optic disk, with the central retinal artery and vein centered on the optic nerve during image acquisition (Figures 1A,B, images were captured in both transverse and sagittal planes). Three measurements were taken in both the transverse and sagittal planes during each measurement session. The mean value derived solely from the transverse planes was designated as mean ONSD(T), and the overall mean of the transverse and sagittal planes was denoted as mean ONSD. Subsequently, the final average values of ONSD were computed from two ONSD evaluations carried out within the 24-h timeframe.

### CCAU measurements

The CCAU examination was conducted following ONSD measurements by above two operators, utilizing the temporal window on both sides and we also employed the Mindary M9 ultrasound system (Shenzhen, Guangzhou, China), utilizing the L12–4 s probe (4-12 MHz) and the SP5-1 s probe (1-5 MHz) at bedside (Figures 1C,D). This bilateral assessment focused on the MCA, with measurements taken 1–2 cm after intracranial ICA bifurcation (the sampling angle is corrected, less than 15) with the help of the SP5-1 s probe. We recorded bilateral MCA flow velocities: systolic (MCAFVs), mean (MCAFVm), diastolic (MCAFVd) and MCAPI--obtained by the automatic tracking method or the manual tracking method of the spectral Doppler, and invasive MAP recorded simultaneously.

Then, in the process of measurement of the parameters of the extracranial ICA, the probe (L12–4 s) was positioned at a distance of 2 cm above the bifurcation of the common carotid artery (CCA). The sampling angle was carefully corrected to be less than 60̊, ensuring it was as parallel as possible to the long axis of the extracranial ICA. We documented the bilateral ICA flow velocities, including systolic (ICAFVs), mean (ICAFVm), diastolic (ICAFVd), and ICAPI, which was obtained through either the Automatic tracking method or the manual tracking method of the spectral Doppler. The definitive ultrasonic indexes were ascertained by computing the average of the values from both sides. The value of the PI ratio is the ratio of MCAPI to ICAPI.

### Estimated ICP

For formula of estimated ICP (ICPe), initially, noninvasive cerebral perfusion pressure (CPPn) was calculated as $\mathrm{MAP}\times \frac{FVd}{FVm}+$ 14 and then, (ICPe = MAP−CPPn) (14). For patients with DC, there is no definite threshold for intracranial hypertension. We have made classifications according to different cut-off values (15, 20, 22 mmHg).

### Statistical methods

Statistical analyses were conducted utilizing MedCalc Statistical Software version 20.022 (MedCalc Software Ltd., Ostend, Belgium). *p* value <0.05 (two-tailed) was considered to indicate statistical signifcance.

The normality of continuous variables was assessed using the Shapiro–Wilk test. Normally distributed variables were compared using the Student’s *t*-test or one-way analysis of variance. Non-normally distributed variables, expressed as median (interquartile range), were analyzed using the Mann–Whitney *U* test. The correlation between continuous variables was determined with the Pearson correlation coefficient, where ‘strong’ was defined as >0.7–1, ‘moderate’ as 0.5–0.7, ‘weak’ as 0.3–0.5, ‘low weak’ as <0.3. The diagnostic accuracy of noninvasive ICP estimators for intracranial hypertension was evaluated using ROC curves, including the calculation of areas under the curves (AUCs), sensitivity, and specificity. Logistic regression analysis was employed to estimate the AUCs with 95% confidence intervals (CIs) for combinations of non-invasive methods, with intracranial hypertension as the outcome variable. The optimal cut-off values for each and combined methods were identified using Youden’s index to balance sensitivity and specificity. The agreement between ICPi and ICPe was assessed using the Bland–Altman method, with 95% CIs for the prediction interval and bias.

### Sampling test

At least 96 participants were required (1-*β* = 0.9, alpha = 0.05, AUC0 = 0.5, AUC1 = 0.70–0.99 (14–17), Lower to Upper FPR (False Positive Rate) = 0.00–1.00, type of data = continuous, Alternative Hypothesis = two-sided test). The sample size and power were calculated using PASS2023, version 23.0.2 for Windows (NCSS, Kaysville, UT, USA).

## Results

### Interobserver variability

Prior to the study, two operators (Y. Zhu and S. Tang) had evaluated 30 randomly selected patients with TBI. Both operators were well-versed in the ‘CLOSED’ protocol for ONSD measurements and had standardized procedures for the CCAU. The assessments were performed blinded to each other’s results. There was an excellent inter-observer agreement for the measurements of the following indices: intraclass correlation coefficient (ICC) with 95% CI as follows: ONSD (0.944, 0.833–0.969), MCAPI (0.941, 0.895–0.977), PI-ratio (0.897, 0.852–0.929), and MCAFVd (0.874, 0.782–0.961). The two ONSD and CCAU measurements post-DC were completed by these two operators on separate occasions within 24 h.

### Study population

**Figure 2:**
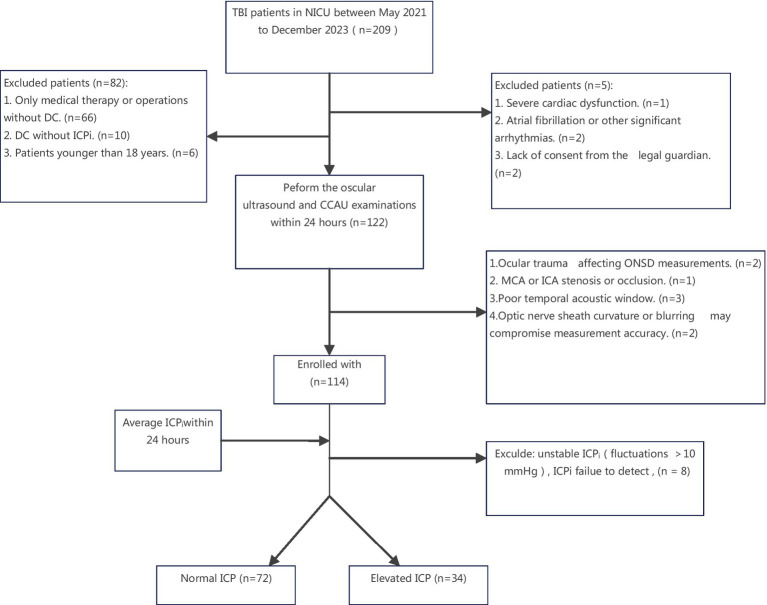
Flowchart of the patient selection process. NICU, Neuro-intensive Care Unit; ICA, internal carotid artery; MCA, middle cerebral artery. ICP, intracranial pressure; CCAU, Cervical-Cerebral Arterial Ultrasound; ONSD, optic nerve sheath diameter; DC, decompressive craniotomy; ICPi, invasive intracranial pressure; TBI, traumatic brain injuries.

A total of 106 TBI patients after DC finally enrolled in our research (Figure 2). Among the patients, 3 received monitoring via a ventricular catheter, while the remaining ones used an intraparenchymal monitor.

The mean ONSD and mean ONSD(T) were all considered for statistical analysis between normal and elevated ICP groups (*p* < 0.05) and their ICC with 95% CI: (0.913, 0.862–0.932). We found that ONSD(T) had a higher measurement success rate and better repeatability compared to ONSD. The highly echogenic margins of the optic nerve sheath were clearly visible in the transverse section. Thus, for the subsequent analysis, we used ONSD(T). MCAPI, PI-ratio, ICPe and MCAFVd demonstrated statistical differences (all *p* < 0.05). Other patient characteristics and evaluated parameters of the study cohort are shown in Table 1.

**Figure 3:**
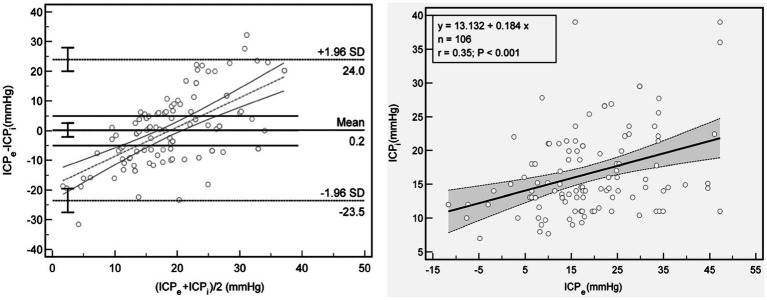
Association between ICPe and ICPi. Left: Bland Altman plot for ICPe compared with ICPi. The mean difference is 0.22 mm Hg, with 95% limits of agreement of −23.5 mm and 24.0 mm Hg (dot lines), and threshold of 5 mmHg (lines). Right: Scatterplot and line regression of ICPe and ICPi. Gray shaded areas on the plots represent 95% CIs for the linear regressions. ICPi, invasive intracranial pressure; ICPe, estimate for ICP; r, Pearson correlation coefficient.

**Figure 4:**
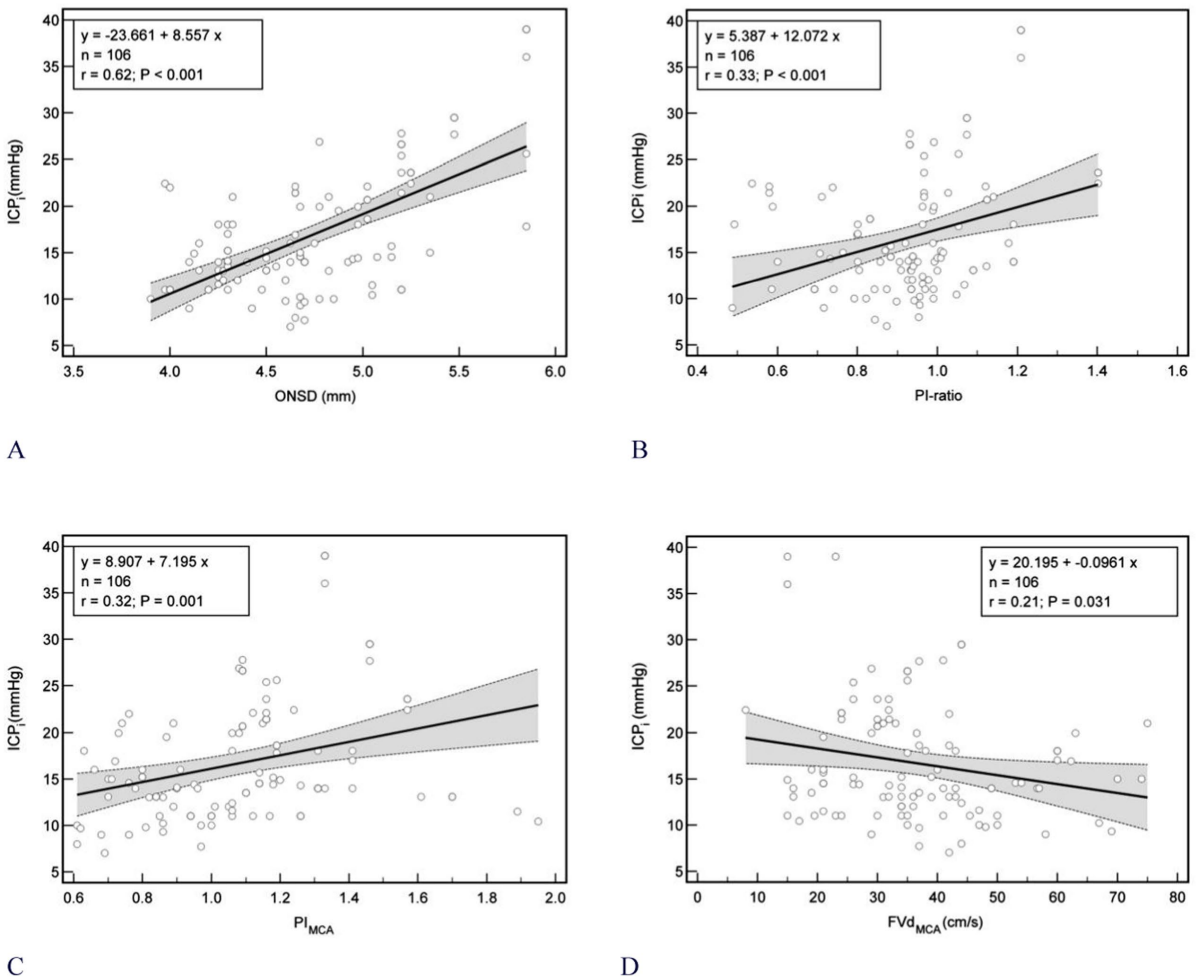
Scatterplot of ICP (mmHg) and different noninvasive ICP estimators between patients (*n* = 106). **(A)** ONSD (*r* = 0.62); **(B)** PI-ratio (*r* = 0.33); **(C)** MCAPI (*r* = 0.32); **(D)** MCAFVd (*r* = 0.21). Gray shaded areas on the plots represent 95% CIs for the linear regressions. ONSD, optic nerve sheath diameter; ICPi_,_ invasive intracranial pressure; PI, pulsatility index; MCAPI_,_ pulsatility index of middle cerebral artery; ICAPI_,_ pulsatility index of internal carotid artery; PI-ratio, MCAPI/ICAPI; ICA, internal carotid artery; MCA, middle cerebral artery. MCAFVd, diastolic flow velocity of MCA; *r*, Pearson correlation coefficient; CIs, confidence intervals.

In the Bland–Altman plot analysis the mean difference between ICPi and ICPe in the overall population was 0.4 mm Hg, with 95% prediction interval (limits of agreement) of −23.5 mm Hg and 24.0 mm Hg (Figure 3 left); Furthermore, in the linear regression model depicted in Figure 3 right and Figures 4A–D, only the ONSD(T) showed a Pearson correlation coefficient (r) of 0.62, which indicates a moderate linear association. In contrast, the other estimators had correlation coefficients below 0.50, suggesting a weak linear association.

The results of the ROC analysis are presented in Table 2 and Supplementary Tables S1–S3. ONSD alone demonstrated the highest AUC among all individual parameters —defined by elevated average ICPi within 24 h exceeding the thresholds of 15 mmHg [AUC: 0.732 (95% CI:0.64–0.81)], 20 mmHg [AUC: 0.74, (95% CI: 0.65–0.82)], 22 mmHg [AUC: 0.781 (95% CI:0.69–0.86)] and elevated simultaneous ICPi threshold of 20 mmHg [AUC: 0.74 (95% CI: 0.65–0.82)]. Under different thresholds of elevated average ICPi, MCAPI obtained the highest NPV among all individual parameter: 15 mmHg [56.8% (95% CI: 47.0–66.1%)], 20 mmHg [92.2% (95% CI: 82.2–96.8%)], 22 mmHg [98.0% (95% CI: 87.9–99.7%)] and its cut-off values are all 1.06. For cutoff values of ONSD(T) and PI-ratio predicting intracranial hypertension, 15 mmHg (4.9 mm, 0.99), 20 mmHg (5.0 mm, 1.01), 22 mmHg (5.0 mm, 1.05), respectively.

**Table 2:** Different non-invasive indexes and their combinations (using logistic regression model) for estimating intracranial hypertension.

Elevated average ICPi within 24 h: thresholds >20 mmHg	ROC related of non-invasive indexes and their combinations								
	Cut off points	Sensitivity, %	Specificity, %	PPV,%	NPV, %	LR+	LR-	AUC	*p* value
PI-ratio	>1.01	55.88 (37.9–72.8)	91.67 (82.7–96.9)	76.0 (58.2–87.8)	81.5 (75.0–86.6)	6.71 (2.95–15.2)	0.48 (0.33–0.71)	0.73 (0.63–0.81)	0.004
ICPe (mmHg)	>17.43	67.65 (49.5–82.6)	58.33 (46.1–69.8)	43.3 (34.8–52.2)	79.3 (69.4–86.6)	1.62 (1.13–2.32)	0.55 (0.33–0.94)	0.631 (0.531–0.722)	0.0228
ONSD (mm)	>5.0	61.76 (43.6–77.8)	90.28 (81.0–96.0)	75.0 (58.6–86.4)	83.3 (76.4–88.5)	6.35 (3.00–13.48)	0.42 (0.27–0.65)	0.74 (0.64–0.82)	0.0002
MCAFVd (cm/s)	≤33	61.76 (43.6–77.8)	67.61 (55.5–78.2)	47.7 (37.3–58.3)	78.7 (70.1–85.4)	1.91 (1.24–2.92)	0.57 (0.36–0.89)	0.62 (0.52–0.72)	0.037
MCAPI	>1.06	88.24 (72.5–96.7)	65.28 (53.1–76.1)	54.5 (46.1–62.8)	**92.2** (82.2–96.8)	2.54 (1.81–3.57)	0.18 (0.071–0.46)	0.74 (0.65–0.82)	<0.0001
PI-ratio + ONSD	>0.41	67.65 (49.5–82.6)	**94.44** (86.4–98.5)	**85.2** (68.3–93.9)	86.1 (79.1–91.0)	12.18 (4.57–32.45)	0.34 (0.21–0.56)	**0.77** (0.68–0.85)	<0.0001
PI-ratio + MCAPI	>0.28	72.41 (52.8–87.3)	80.52 (69.9–88.7)	58.3 (45.8–69.9)	88.6 (81.0–93.4)	3.72 (2.24–6.17)	0.34 (0.19–0.62)	0.74 (0.64–0.82)	0.0002
ONSD+MCAPI	>0.36	67.65 (49.5–82.6)	79.17 (68.0–87.8)	57.1 (44.3–69.1)	87.3 (79.8–92.3)	3.25 (1.96–5.39)	0.41 (0.25–0.67)	0.76 (0.67–0.84)	<0.0001
PI-ratio + ONSD +MCAPI+MCAFVd	>0.38	67.65 (49.5–82.6)	85.92 (75.6–93.0)	63.9 (50.7–75.3)	84.1 (76.2–89.7)	3.69 (2.15–6.36)	0.40 (0.24–0.65)	0.76 (0.67–0.84)	< 0.0001

PPV, positive predictive value; NPV, negative predictive value; LR+, postive likelihood ratio; LR-, negative likelihood ratio; AUC, area under the curve. ROC, receiver operator characteristic curves; ONSD, optic nerve sheath diameter; ICP, intracranial pressure; MCAPI, pulsatility index of middle cerebral artery; ICAPI, pulsatility index of internal carotid artery; PI-ratio, MCAPI/ICAPI; MCAFVd, diastolic flow velocity of middle cerebral artery.

Under all cut-off values, the highest specificity and PPV were obtained by the combined parameters (ONSD(T) + PI-ratio), respectively: 15 mmHg [92.86% (95% CI:80.5–98.5%), 91.3% (95% CI:77.2–96.9%)], 20 mmHg [94.44% (95% CI:86.4–98.5%), 85.2% (95% CI:68.3–93.9%)], 22 mmHg [87.95% (95% CI:79.0–94.1%), 61.5% (95% CI:45.7–75.2%)]. This combined approach also attains the highest AUCs. Nevertheless, in comparison with the ONSD(T) alone, it fails to exhibit a statistically significant enhancement in AUC values (*p* > 0.05, as per DeLong’s test).

## Discussion

In this prospective, single-center investigation, we discovered that MCAPI could reliably rule out intracranial hypertension in TBI patients following DC. Among the cases identified as having normal ICP by MCAPI, 92% had a normal ICPi when a threshold of >20 mmHg was employed, 98% with a threshold >22 mmHg, yet only 57% with a threshold of >15 mmHg. Our findings suggest that the application of TCCD might be beneficial for patients with moderate to severe intracranial hypertension (ICP > 20 mmHg) in situations where invasive ICP measurement is either unavailable or impractable.

We also ascertain that ICPe fails to precisely estimate ICPi within TBI patients with DC (Figure 3). Although a weak correlation exists between ICPe and ICPi, the margin of error is extremely large, which is consistent with the results of previous studies (18). Simultaneously, ICPe is demonstrated to be an ineffective screening test for intracranial hypertension, sharing certain similarities with the prospective multicenter international study by Rasulo et al. (19, 20). However, the key difference lies in the fact that our study reveals it is not a reliable exclusion test for patients with DC. Potentially, this is due to the disruption of the Monroe-Kelly doctrine (After DC, the contents within the cranial cavity can bulge toward the decompressive bone window. This modification not only disrupts the original equilibrium state but also impacts cerebrovascular autoregulation. Consequently, the traditional pressure-regulating mechanism, which is premised on a closed cranial cavity, is affected.), which renders the ICPe formula inapplicable in light of these alterations.

On the other hand, we initially introduced the concept of CCAU in our experiment. A series of statistical analyses were conducted on its associated parameters. Parameters like FVs-ratio (MCAFVs/MCAFVs), FVm-ratio (MCAFVm/ICAFVm), and FVd-ratio (MCAFVd/ICAFVd) did not display any statistical significance. Only PI-ratio, MCAPI, MCAFVd show distinct results in DC patients experiencing intracranial hypertension. Additionally, the correlations between these metrics and ICPi were found to be weak, and this weakness was even more evident when compared to the correlations obtained by ONSD following the CLOSED protocol. These results align with those of some prior studies, where the r between PI and ICP was 0.31 (18, 21). Although certain scholars (22, 23) have claimed a strong association between MCAPI, MCAFVd, and ICP, it is widely recognized that the accuracy of this method can be significantly influenced by a host of external factors, such as MAP, PaO2, PaCO2, and cerebral autoregulation of blood flow, especially in DC patients. When solely comparing the PI-ratio with MCAPI, across different thresholds of intracranial hypertension, the PI-ratio, serving as an individual predictive metric, proved to be at least on a par with MCAPI. Notably, when it was paired with ONSD, it attained the optimal AUCs. This result thereby validates our previous theoretical basis.

To the best of our knowledge, there have been no reports on the application of CCAU in non-invasive ICP monitoring for post-DC patients. In contrast to TCD, CCAU offers distinct advantages, including visualization, angle adjustability, as well as standardized and convenient operating procedures, which make it highly suitable for rapid ICP assessment in emergency scenarios. Nevertheless, the practical value of CCAU still awaits further evaluation and exploration through multi-center, large-scale studies.

ONSD monitoring is another emerging non-invasive technique for assessing ICP, capitalizing on the fact that increases in ICP are transmitted through the subarachnoid space, which in turn leads to an enlargement of the ONSD (14, 24). In our research, ONSD performs best among metircs, with optimal cutoffs of 4.9 mm, 5.0 mm, 5.0 mm at ICPi thresholds of 15 mmHg, 20 mmHg, 22 mmHg, consistent with prior studies (25, 26). Under the CLOSED protocol, results have excellent inter-observer agreement (ICC: 0.944) and good application value (AUC: 0.74) for post-DC patients. However, ONSD currently has drawbacks that limit its wider use. Its measurement range is broad across healthy and sick populations, diagnostic thresholds vary, and factors like age, gender, ethnicity come into play (27). Also, some studies use non-standard methods, wrongly measuring outer margins, yielding false, inflated values (28, 29). Consequently, the utilization of parameters from ONSD or CCAU for gaging ICP must be exercised with discretion.

As previously noted, the Monroe-Kelly doctrine no longer holds for DC patients, given their unique condition. Even when ICPi lies within the normal range post-DC, the presence of brain tissue edema cannot be excluded (30). Consequently, in some DC patients, despite a normal ICPi, the measured values of CCAU and ONSD may already be elevated. This accounts for the relatively high specificity (92.86%) of the ONSD and PI-ratio combination in patients with a mild ICPi elevation (> 15 mmHg) in our experiment. For patients showing increased ONSD and PI-ratio values, prompt interventions, including ICP reduction treatments, emergency CT, or MRI scans, should be carried out.

At last but not the least, although ONSD and CCAU can not facilitate continuous readings, they could be conducted by one operator simultaneously and recognized for its expediency, safety, and reproducibility, without the need for specialized software. Additionally, the accessibility of ultrasound equipment in the majority of NICUs and other clinical settings allows healthcare providers to evaluate cerebrovascular dynamics without necessitating the transfer of patients to the neuroradiology department. The appeal of ultrasound-based measurement techniques is heightened when invasive monitoring is not feasible or when there is a suggestion for monitoring that does not reach the level of a strong indication.

### Limitations

Several limitations within our study warrant consideration. First, although the noninvasive approach allowed for quick and repeatable assessments, the CCAU and ONSD measurements were subject to subjectivity and were not performed continuously. This could introduce the potential for operator bias, particularly with multiple measurements taken at different time points. To minimize this bias, we adopted an approach where we performed twice evaluation of CCAU and ONSD within the first 24 h with different operators and aimed to keep patients’ ICP fluctuations within a maximum of 10 mmHg over this period, which, while sometimes restrictive in a clinical context, was deemed necessary. Second, the constrained sample size limited our capacity to detect differences, underscoring the need for future multicenter studies with larger cohorts. Third, unlike regular TBI cases, TBI patients post-DC may not adhere to the Monroe-Kelly doctrine, and their ICP thresholds are less precisely defined. Potentially, more parameters such as the size of the surgical incision, the duration of the operation, and the amount of blood loss are required for classification and evaluation. Fourth, the incidence of intracranial hypertension (ICP < 20 mmHg, 32%) was lower than in other series, potentially limiting result generalization to populations with more severe ICP patients. Fifth, during critical illness, extracranial factors (e.g., massive visceral hemorrhage, multiple fractures, deep vein thrombosis) may cause hemodynamic instability, affecting the correlation between true ICPi and relevant parameters (e.g., TCCD or CCAU) and likely leading to dissociation. Sixth, as per the study design, patient outcomes were not assessed, so the impact of using these non-invasive monitorings on morbidity and mortality remains unknown. Finally, the study was limited to observing the correlation between ONSD, ICP, and CCAU parameters, without extending to the assessment of cerebral metabolic and oxygenation statuses or the inclusion of prognostic evaluations for the patients.

## Conclusion

ICPe, MCAPI, MCAFVd, Ultrasonographic ONSD and PI-ratio demonstrate a low weak to moderate correlation with ICPi. ICPe alone is not considered sufficiently precise for noninvasive ICP assessment. The concurrent utilization of CCAU and ONSD measurements based on CLOSED protocol may offer superior accuracy in diagnosis for early elevated ICP (< 24 h) in TBI patients post-DC. Under different thresholds of intracranial hypertension, MCAPI obtained the highest NPV and was the most sensitive single index in excluding intracranial hypertension in our study. Further research is imperative to validate these findings within a more extensive patient population.

## Data Availability

The raw data supporting the conclusions of this article will be made available by the authors, without undue reservation.

## References

[1] Faria BCD Sacramento LGG Queiroz AVR Leite FAD Oliveira H Kimura TY. The use of noninvasive measurements of intracranial pressure in patients with traumatic brain injury: a narrative review. Arq Neuropsiquiatr. (2023) 81:551–63. doi: 10.1055/s-0043-1764411, PMID: 37379867 PMC10306993

[2] Harary M Dolmans RGF Gormley WB. Intracranial pressure monitoring-review and avenues for development. Sensors. (2018) 18:465. doi: 10.3390/s18020465, PMID: 29401746 PMC5855101

[3] Roldan M Abay TY Kyriacou PA. Non-invasive techniques for multimodal monitoring in traumatic brain injury: systematic review and Meta-analysis. J Neurotrauma. (2020) 37:2445–53. doi: 10.1089/neu.2020.7266, PMID: 32821023

[4] Demetriades AK. Intracranial pressure monitoring after primary decompressive craniectomy: is it useful? Acta Neurochir. (2017) 159:623–4. doi: 10.1007/s00701-017-3119-y, PMID: 28243808

[5] Picetti E Caspani ML Iaccarino C Pastorello G Salsi P Viaroli E. Intracranial pressure monitoring after primary decompressive craniectomy in traumatic brain injury: a clinical study. Acta Neurochir. (2017) 159:615–22. doi: 10.1007/s00701-017-3118-z, PMID: 28236181

[6] Chesnut RM Temkin N Carney N Dikmen S Rondina C Videtta W. A trial of intracranial-pressure monitoring in traumatic brain injury. N Engl J Med. (2012) 367:2471–81. doi: 10.1056/NEJMoa1207363, PMID: 23234472 PMC3565432

[7] Melhem S Shutter L Kaynar A. A trial of intracranial pressure monitoring in traumatic brain injury. Crit Care. (2014) 18:302. doi: 10.1186/cc13713, PMID: 24485039 PMC4056074

[8] Murillo-Cabezas F Godoy DA. Intracranial pressure monitoring in severe traumatic brain injury: a different perspective of the BestTrip trial. Med Intensiva. (2014) 38:237–9. doi: 10.1016/j.medin.2013.07.011, PMID: 24674667

[9] Stevens AR Su Z Toman E Belli A Davies D. Optical pupillometry in traumatic brain injury: neurological pupil index and its relationship with intracranial pressure through significant event analysis. Brain Inj. (2019) 33:1032–8. doi: 10.1080/02699052.2019.1605621, PMID: 31021683

[10] Robba C Pozzebon S Moro B Vincent JL Creteur J Taccone FS. Multimodal non-invasive assessment of intracranial hypertension: an observational study. Crit Care. (2020) 24:379. doi: 10.1186/s13054-020-03105-z, PMID: 32591024 PMC7318399

[11] Nucci CG De Bonis P Mangiola A Santini P Sciandrone M Risi A. Intracranial pressure wave morphological classification: automated analysis and clinical validation. Acta Neurochir. (2016) 158:581–8. doi: 10.1007/s00701-015-2672-5, PMID: 26743919

[12] Singer KE Wallen TE Jalbert T Wakefield D Spuzzillo A Sharma S. Efficacy of noninvasive Technologies in Triaging Traumatic Brain Injury and Correlating with Intracranial Pressure: a prospective study. J Surg Res. (2021) 262:27–37. doi: 10.1016/j.jss.2020.12.042, PMID: 33540153

[13] Aspide R Bertolini G Albini Riccioli L Mazzatenta D Palandri G Biasucci DG. A proposal for a new protocol for sonographic assessment of the optic nerve sheath diameter: the CLOSED protocol. Neurocrit Care. (2020) 32:327–32. doi: 10.1007/s12028-019-00853-x, PMID: 31583527

[14] Raffiz M Abdullah JM. Optic nerve sheath diameter measurement: a means of detecting raised ICP in adult traumatic and non-traumatic neurosurgical patients. Am J Emerg Med. (2017) 35:150–3. doi: 10.1016/j.ajem.2016.09.044, PMID: 27852525

[15] Robba C Cardim D Tajsic T Pietersen J Bulman M Donnelly J. Ultrasound non-invasive measurement of intracranial pressure in neurointensive care: a prospective observational study. PLoS Med. (2017) 14:e1002356. doi: 10.1371/journal.pmed.1002356, PMID: 28742869 PMC5526499

[16] Maissan IM Dirven PJ Haitsma IK Hoeks SE Gommers D Stolker RJ. Ultrasonographic measured optic nerve sheath diameter as an accurate and quick monitor for changes in intracranial pressure. J Neurosurg. (2015) 123:743–7. doi: 10.3171/2014.10.JNS141197, PMID: 25955869

[17] Czosnyka M Matta BF Smielewski P Kirkpatrick PJ Pickard JD. Cerebral perfusion pressure in head-injured patients: a noninvasive assessment using transcranial Doppler ultrasonography. J Neurosurg. (1998) 88:802–8. doi: 10.3171/jns.1998.88.5.0802, PMID: 9576246

[18] Cardim D Robba C Bohdanowicz M Donnelly J Cabella B Liu X. Non-invasive monitoring of intracranial pressure using transcranial Doppler ultrasonography: is it possible? Neurocrit Care. (2016) 25:473–91. doi: 10.1007/s12028-016-0258-6, PMID: 26940914 PMC5138275

[19] Rasulo FA Calza S Robba C Taccone FS Biasucci DG Badenes R. Transcranial Doppler as a screening test to exclude intracranial hypertension in brain-injured patients: the IMPRESSIT-2 prospective multicenter international study. Crit Care. (2022) 26:110. doi: 10.1186/s13054-022-03978-2, PMID: 35428353 PMC9012252

[20] Rasulo FA Bertuetti R Robba C Lusenti F Cantoni A Bernini M. The accuracy of transcranial Doppler in excluding intracranial hypertension following acute brain injury: a multicenter prospective pilot study. Crit Care. (2017) 21:44. doi: 10.1186/s13054-017-1632-2, PMID: 28241847 PMC5329967

[21] Zweifel C Czosnyka M Carrera E de Riva N Pickard JD Smielewski P. Reliability of the blood flow velocity pulsatility index for assessment of intracranial and cerebral perfusion pressures in head-injured patients. Neurosurgery. (2012) 71:853–61. doi: 10.1227/NEU.0b013e3182675b42, PMID: 22791038

[22] Bellner J Romner B Reinstrup P Kristiansson KA Ryding E Brandt L. Transcranial Doppler sonography pulsatility index (PI) reflects intracranial pressure (ICP). Surg Neurol. (2004) 62:45–51. doi: 10.1016/j.surneu.2003.12.007 15226070

[23] Schmidt B Czosnyka M Raabe A Yahya H Schwarze JJ Sackerer D. Adaptive noninvasive assessment of intracranial pressure and cerebral autoregulation. Stroke. (2003) 34:84–9. doi: 10.1161/01.STR.0000047849.01376.AE, PMID: 12511755

[24] Liu D Li Z Zhang X Zhao L Jia J Sun F. Assessment of intracranial pressure with ultrasonographic retrobulbar optic nerve sheath diameter measurement. BMC Neurol. (2017) 17:188. doi: 10.1186/s12883-017-0964-5, PMID: 28962603 PMC5622417

[25] Rajajee V Vanaman M Fletcher JJ Jacobs TL. Optic nerve ultrasound for the detection of raised intracranial pressure. Neurocrit Care. (2011) 15:506–15. doi: 10.1007/s12028-011-9606-8, PMID: 21769456

[26] Soliman I Johnson G Gillman LM Zeiler FA Faqihi F Aletreby WT. New optic nerve sonography quality criteria in the diagnostic evaluation of traumatic brain injury. Crit Care Res Pract. (2018) 2018:1–7. doi: 10.1155/2018/3589762, PMID: 29854448 PMC5952494

[27] Lochner P Czosnyka M Naldi A Lyros E Pelosi P Mathur S. Optic nerve sheath diameter: present and future perspectives for neurologists and critical care physicians. Neurol Sci. (2019) 40:2447–57. doi: 10.1007/s10072-019-04015-x, PMID: 31367861

[28] Krogias C Ayzenberg I Schroeder C Gruter T Gold R Yoon MS. Transorbital sonography in CIDP patients: no evidence for optic nerve hypertrophy. J Neurol Sci. (2016) 362:206–8. doi: 10.1016/j.jns.2016.01.049, PMID: 26944149

[29] Topcuoglu MA Arsava EM Bas DF Kozak HH. Transorbital Ultrasonographic measurement of optic nerve sheath diameter in brain death. J Neuroimaging. (2015) 25:906–9. doi: 10.1111/jon.12233, PMID: 25800801

[30] Wang J Li K Li H Ji C Wu Z Chen H. Ultrasonographic optic nerve sheath diameter correlation with ICP and accuracy as a tool for noninvasive surrogate ICP measurement in patients with decompressive craniotomy. J Neurosurg. (2020) 133:514–20. doi: 10.3171/2019.4.JNS183297, PMID: 31323632

