# Supplementary material for: Post-craniotomy intracranial pressure monitoring: a novel approach combining optic nerve sheath diameter ultrasonography and cervical-cerebral arterial ultrasound
Source: Front Neurol. 2025 Jan 15;15:1472494. doi: 10.3389/fneur.2024.1472494 (PMC11774741; doi:10.3389/fneur.2024.1472494)
Supplement: Supplementary file 1 [file Table_1.DOCX]

**Table S1 (supplementary 1).** Different non-invasive indexes and their combinations (using logistic regression model) for estimating intracranial hypertension

| Elevated average ICPi within 24 hours: thresholds > 15 mmHg | ROC related of non-invasive indexes and their combinations | | | | | | | | |
| --- | --- | --- | --- | --- | --- | --- | --- | --- | --- |
|  | Cut off points | Sensitivity, % | Specificity, % | PPV,% | NPV, % | LR+ | LR- | AUC | *P* value |
| PI-ratio | ＞  0.99 | 43.75(31.4 - 56.7) | 88.10(74.4 - 96.0) | 84.9  (70.2 - 93.0) | 50.7  (44.6 - 56.7) | 3.67  (1.54 - 8.76) | 0.64 (0.50 - 0.81) | 0.659 (0.560-0.748) | 0.0025 |
| ICPe(mmHg) | >21.03 | 51.56  (38.7 - 64.2) | 76.19  (60.5 - 87.9) | 76.7  (64.6 - 85.6) | 50.8  (43.2 - 58.3) | 2.17  (1.20 - 3.91) | 0.64  (0.47 - 0.86) | 0.608(0.508 -0.701) | 0.0574 |
| ONSD(mm) | ＞4.9 | 51.56 (38.7 - 64.2) | 88.10 (74.4 - 96.0) | 86.9  (73.7 - 94.0) | 54.4  (47.5 - 61.1） | 4.33 (1.84 - 10.20) | 0.55 (0.42-0.72) | **0.732** (0.638-0.814) | ＜0.001 |
| MCAFVd (cm/s） | ≤ 33 | 49.21  （36.4 - 62.1） | 69.05  （52.9 - 82.4） | 70.8  （59.1 - 80.3） | 47.1  （39.4 - 55.0） | 1.59  （0.95 - 2.67） | 0.74  （0.54 - 1.01） | 0.566 （0.466-0.663） | 0.264 |
| MCAPI | >1.06 | 65.62  (52.7 - 77.1) | 69.05  (52.9 - 82.4) | 76.4  (66.6 - 84.0) | **56.8**  (47.0 - 66.1) | 2.12  （1.31 - 3.44） | 0.50  （0.34 - 0.74） | 0.625（0.526 to 0.717） | 0.0298 |
| PI-ratio+ONSD | >0.725 | 48.44  35.8 - 61.3 | **92.86**  (80.5 - 98.5) | **91.3**  (77.2 - 96.9) | 54.1  (47.9 - 60.3) | 6.78  2.21 - 20.77 | 0.56  0.43 - 0.71 | 0.743 (0.649 to 0.823) | ＜0.001 |
| PI-ratio+MCAPI | >0.639 | 48.44  (35.8 - 61.3) | 85.71  (71.5 - 94.6) | 84.2  (71.0 - 92.1) | 52.9  (46.1 - 59.7) | 3.39  (1.55 - 7.42) | 0.60  (0.46 - 0.79) | 0.667(0.569 to 0.756) | 0.0016 |
| ONSD+MCAPI | >0.726 | 50.00  (37.2 - 62.8) | 90.48  (77.4 - 97.3) | 88.9  (75.3 - 95.5) | 54.3  (47.7 - 60.7) | 5.25  (2.00 - 13.76) | 0.55  (0.42 - 0.72) | 0.734(0.640 to 0.815) | < 0.0001 |
| PI-ratio +ONSD +MCAPI+MCAFVd | >0.706 | 49.21  (36.4 - 62.1) | **92.86**  (80.5 - 98.5) | **91.3**  (77.4 - 97.0) | 54.5  (48.1 - 60.8) | 6.89  (2.25 - 21.09) | 0.55  (0.42 - 0.71) |  | < 0.0001 |

**Table S2 (supplementary 2).** Different non-invasive indexes and their combinations (using logistic regression model) for estimating intracranial hypertension

| Elevated average ICPi within 24 hours: thresholds > 22 mmHg | ROC related of non-invasive indexes and their combinations | | | | | | | | |
| --- | --- | --- | --- | --- | --- | --- | --- | --- | --- |
|  | Cut off points | Sensitivity, % | Specificity, % | PPV,% | NPV, % | LR+ | LR- | AUC | *P* value |
| PI-ratio | >1.05 | 47.83  (26.8 - 69.4) | 85.54  (76.1 - 92.3) | 47.8  (31.8 - 64.3) | 85.5  (79.8 - 89.8) | 3.31  (1.68 - 6.50) | 0.61  (0.41 - 0.91) | 0.649(0.551 - 0.739) | 0.052 |
| ICPe(mmHg) | >25.86 | 56.52  (34.5- 76.8) | 83.13  (73.3 - 90.5) | 48.2  (33.8-62.8) | 87.3  (81.1-91.7) | 3.35  (1.84 -6.09) | 0.52  (0.32 -0.84) | 0.719(0.623 to 0.802) | 0.0003 |
| ONSD(mm) | >5 | 69.57  (47.1 - 86.8) | 85.54  (76.1 - 92.3) | 57.1  (42.5 - 70.6) | 91.0  (84.5 - 95.0) | 4.81  (2.67 - 8.67) | 0.36  (0.19 - 0.66) | **0.781**(0.691 -0.856) | < 0.0001 |
| MCAFVd (cm/s） | ≤44 | 91.30  (72.0 - 98.9) | 30.49  (20.8 - 41.6) | 26.7  (23.1 - 30.6) | 92.7  (76.4 - 98.0) | 1.31  (1.09 - 1.59) | 0.29  (0.073 - 1.12) | 0.658(0.559 - 0.748) | 0.0068 |
| MCAPI | >1.06 | 95.65  (78.1 - 99.9) | 60.24  (48.9 - 70.8) | 40.0  (33.5 - 46.8) | **98.0**  (87.9 - 99.7) | 2.41  (1.82 - 3.18) | 0.072  (0.011 - 0.49) | 0.769 (0.677 to 0.845) | < 0.0001 |
| PI-ratio+ONSD | >0.274 | 69.57  (47.1 - 86.8) | **87.95**  (79.0 - 94.1) | **61.5**  (45.7 - 75.2) | 91.2  (84.8 - 95.1) | 5.77  (3.04 - 10.96) | 0.35  (0.19 - 0.65) | 0.786((0.696 -0.860) | <0.0001 |
| PI-ratio+MCAPI | >0.191 | 82.61  (61.2 - 95.0) | 61.45  (50.1 - 71.9) | 37.3  (29.9 - 45.2) | 92.7  (83.7 - 96.9) | 2.14  (1.54 - 2.98) | 0.28  (0.11 - 0.70) | 0.750 (0.656 -0.829) | <0.0001 |
| ONSD+MCAPI | >0.233 | 78.26  (56.3 - 92.5) | 79.52  (69.2 - 87.6) | 51.4  (39.7 - 63.0) | 93.0  (85.8 - 96.7) | 3.82  (2.38 - 6.15) | 0.27  (0.12 - 0.60) | 0.792((0.703 to 0.865) | <0.0001 |
| PI-ratio +ONSD +MCAPI+MCAFVd | >0.238 | 86.96  (66.4 - 97.2) | 79.27  (68.9 - 87.4) | 53.8  (42.5 - 64.6) | 95.6  (88.4 - 98.4) | 4.19  (2.67 - 6.59) | 0.16  (0.057 - 0.48) | 0.841(0.757 to 0.905) | <0.0001 |

**Table S3 (supplementary 3).** Different non-invasive indexes and their combinations (using logistic regression model) for estimating intracranial hypertension

| Elevated simultaneous ICPi thresholds: > 20 mmHg | ROC related of non-invasive indexes and their combinations | | | | | | | | |
| --- | --- | --- | --- | --- | --- | --- | --- | --- | --- |
|  | Cut off points | Sensitivity, % | Specificity, % | PPV,% | NPV, % | LR+ | LR- | AUC | ***P*** value |
| PI-ratio | ＞0.96 | 72.41（52.8 - 87.3） | 71.43（60.0 - 81.2） | 48.8（38.6 - 59.2） | 87.3（78.9 - 92.7） | 2.53（1.67 - 3.85） | 0.3（0.21 - 0.719） | 0.72（0.62-0.80） | 0.0012 |
| ICPe(mmHg) | >21.41 | 58.62  38.9 - 76.5 | 68.83  57.3 - 78.9 | 41.5  31.1 - 52.6 | 81.5  73.6 - 87.5 | 1.88  1.20 - 2.95 | 0.60  0.38 - 0.95 | 0.658(0.561 to 0.748) | 0.006 |
| ONSD(mm) | ＞4.9 | 72.41（52.8 - 87.3） | 77.92（67.0 - 86.6） | 55.3（43.4 - 66.5） | 88.2（80.4 - 93.2） | 3.28（2.04 - 5.28） | 0.35（0.19 - 0.65） | **0.74**（0.65-0.82） | 0.0002 |
| MCAFVd (cm/s） | ≤33 | 68.97（49.2 - 84.7） | 68.42（56.7 - 78.6） | 45.5（35.6 - 55.7） | 85.2（76.7 - 91.0） | 2.18（1.45 - 3.29） | 0.45（0.26 - 0.80） | 0.67 (0.58-0.76) | 0.0018 |
| MCAPI | ＞1.06 | 86.21（68.3 - 96.1） | 61.04（49.2 - 72.0） | 45.5（37.8 - 53.3） | **92.2**（82.3 - 96.7） | 2.21（1.61 - 3.03） | 0.23（0.089 - 0.57） | 0.68(0.59-0.77) | 0.0012 |
| PI-ratio+ONSD | ＞0.64 | 69.1(49.2-84.7) | **94.81**(87.2-98.6) | **83.3**(65.1 - 93.0) | 89.0(82.5 - 93.3) | 13.28 (4.96 - 35.55) | 0.33 (0.19 - 0.56) | **0.77**(0.69-0.85) | < 0.0001 |
| PI-ratio+MCAPI | ＞0.28 | 72.41（52.8 - 87.3） | 80.52（69.9 - 88.7） | 58.3（45.8 - 69.9） | 88.6（81.0 - 93.4） | 3.72（2.24 - 6.17） | 0.34（0.19 - 0.62） | 0.74（0.64-0.82） | 0.0002 |
| ONSD+MCAPI | ＞0.31 | 68.97（49.2 - 84.7） | 80.52（69.9 - 88.7） | 57.1（44.3 - 69.1） | 87.3（79.8 - 92.3） | 3.54（2.11 - 5.93） | 0.39（0.22 - 0.67） | 0.75（0.65-0.83） | 0.0001 |
| PI-ratio +ONSD +MCAPI+MCAFVd | >0.31 | 72.41（52.8 - 87.3） | 78.95（68.1 - 87.5） | 56.8（44.6 - 68.2） | 88.2（80.4 - 93.2） | 3.44（2.11 - 5.61） | 0.35（0.19 - 0.64） | 0.77(0.68-0.85) | < 0.0001 |

PPV, positive predictive value; NPV, negative predictive value; LR+, postive likelihood ratio; LR-, negative likelihood ratio; AUC, area under the curve. ROC : receiver operator characteristic curves; ONSD, optic nerve sheath diameter; ICP, intracranial pressure; MCAPI, pulsatility index of middle cerebral artery; ICAPI, pulsatility index of internal carotid artery; PI-ratio, MCAPI/ICAPI; MCAFVd : diastolic flow velocity of middle cerebral artery;
